# Temporal and spatial distribution of anthrax outbreaks among Kenyan wildlife, 1999–2017

**DOI:** 10.1017/S0950268819001304

**Published:** 2019-08-05

**Authors:** J. M. Gachohi, F. Gakuya, I. Lekolool, E. Osoro, L. Nderitu, P. Munyua, I. Ngere, N. Kemunto, B. Bett, F. Otieno, M. Muturi, A. Mwatondo, M. A. Widdowson, M. Kariuki Njenga

**Affiliations:** 1 Washington State University Global Health Kenya, Washington State University, Nairobi, Kenya; 2 School of Public Health, Jomo Kenyatta University of Agriculture and Technology, Nairobi, Kenya; 3 Kenya Wildlife Service, Nairobi, Kenya; 4 Division of Global Health Protection, United States Centers for Disease Control and Prevention, Nairobi, Kenya; 5 International Livestock Research Institute, Nairobi, Kenya; 6 Kenya Zoonotic Disease Unit, Nairobi, Kenya

**Keywords:** Anthrax, Kenya, outbreaks, wildlife

## Abstract

The burden of anthrax in wildlife is demonstrated through high numbers of sudden mortalities among herbivore species, including endangered animal species. East Africa is home of multiple species of faunal wildlife numbering in the millions but there are limited disease surveillance programmes, resulting in a paucity of information on the role of anthrax and other infectious diseases on declining wildlife populations in the region. We reviewed historical data on anthrax outbreaks from Kenya Wildlife Service (KWS) spanning from 1999 to 2017 in Kenya to determine the burden, characteristics and spatial distribution of anthrax outbreaks. A total of 51 anthrax outbreaks associated with 1014 animal deaths were reported across 20 of 60 wildlife conservation areas located in six of the seven agro-ecological zones. Overall, 67% of the outbreaks were reported during the dry seasons, affecting 24 different wildlife species. Over 90% (22 of 24) of the affected species were herbivore, including 12 grazers, five browsers and five mixed grazers and browsers. Buffaloes (23.5%), black rhinos (21.6%) and elephants (17.6%) were the most frequently affected species. Our findings demonstrate the extensive geographic distribution of wildlife anthrax in the country, making it one of the important infectious diseases that threaten wildlife conservation.

## Introduction

The high loss of faunal diversity in national parks and a growing number of endangered wildlife species have highlighted the need for an improved wildlife disease surveillance in order to identify prevalent diseases for prevention and control [1, 2]. Enhanced and timely wildlife disease surveillance and subsequent control and prevention of some of these diseases would also prevent spill over of endemic and emerging diseases to livestock and humans, supporting global health security [1, 3]. However, wildlife surveillance presents unique challenges associated with limited access to animals, case identification and ecological characteristics of national parks and other forested areas where wildlife inhabit, such as expansiveness of conservation areas [4]. In sub-Saharan Africa, wildlife disease surveillance systems are limited to responding to large outbreaks or narrowly focused retrospective and prospective studies, approaches that have helped in implementing disease outbreak control strategies, estimating specific disease burden and identifying wildlife reservoirs of livestock or human diseases [5–8].

Anthrax, a highly infectious disease of domestic and wild mammals caused by *Bacillus anthracis* bacteria, can result in a large-scale loss of wildlife and livestock worldwide [5–8]. For example, an anthrax outbreak in Luangwa river valley in Zambia resulted in the death of >4000 hippopotami in addition to buffalos and elephants [6]. Another outbreak in Malilangwe wildlife reserve in Zimbabwe resulted in the death of almost all the kudu, and 40–70% of bushbucks, waterbucks, nyala, buffalos and roan antelopes [7]. Between October 2001 and June 2002, an anthrax outbreak in the tropical forest of Ivory Coast resulted in the death of eight chimpanzees [5]. In fact, a recent comprehensive study of this tropical rain forest found that *B. anthracis* has been responsible for widespread and persistent mortality among domestic and wild mammals for three decades [8]. Even in developed countries such as Canada, substantial death of wild bison associated with anthrax outbreaks has been reported [9]. The East African region consists of one of the largest and most diverse wildlife ecosystems in the world, in particular the contiguous ecosystem of Masai Mara and Amboseli national parks in Kenya, and Serengeti and Kilimanjaro national parks in Tanzania [10]. Furthermore, the world-famous annual wildebeest (*Connochaetes taurinus*) migration occurs between the Masai Mara and Serengeti national parks [11]. A recent study in Kenya reported three anthrax outbreaks over a period of 4 years in the same locality of Rift valley in Kenya that resulted in the death of 10.5% of all wildlife herbivores in a local national park, including 17% of the buffalos and 16% endangered black rhinos and white rhinos [12].

Here, we reviewed wildlife records to describe the temporal and spatial distribution of anthrax outbreaks among Kenyan wildlife between 1999 and 2017, a period when there was an improved collection of wildlife disease data in the country. This improvement was catalysed by establishing the One Health office in Kenya of which the Kenya Wildlife Services (KWS) participated in its creation since 2011. The office is operationalised through a zoonotic disease unit (ZDU) with staff drawn from Kenya's Ministries of Health and that of Agriculture, Livestock and Fisheries [13]. Since its operational launch on 1 March 2012, the need for effective wildlife health surveillance has been progressively recognised within KWS to align with ZDU's objective of instituting and maintaining active collaboration at the animal, human and environment nexus focused on better prevention and control of zoonotic diseases.

## Methods

We reviewed records at the KWS of diagnosed anthrax outbreaks and occurrences in wildlife in Kenya from 1999 to 2017. Records included annual reports, specific case reports, surveillance reports and post-mortem reports. At the KWS, the anthrax disease records were generated from clinical surveillance of dead or visibly sick animals by the wildlife veterinarians and game rangers. A confirmed anthrax outbreak was defined as one in which at least one case was diagnosed using Gram and methylene blue stains and identification of the capsule and typical rod-shaped *B. anthracis* in clinical specimens submitted to the KWS laboratory in Nairobi or one of the regional veterinary investigation laboratories [14].

**Fig. 1.**
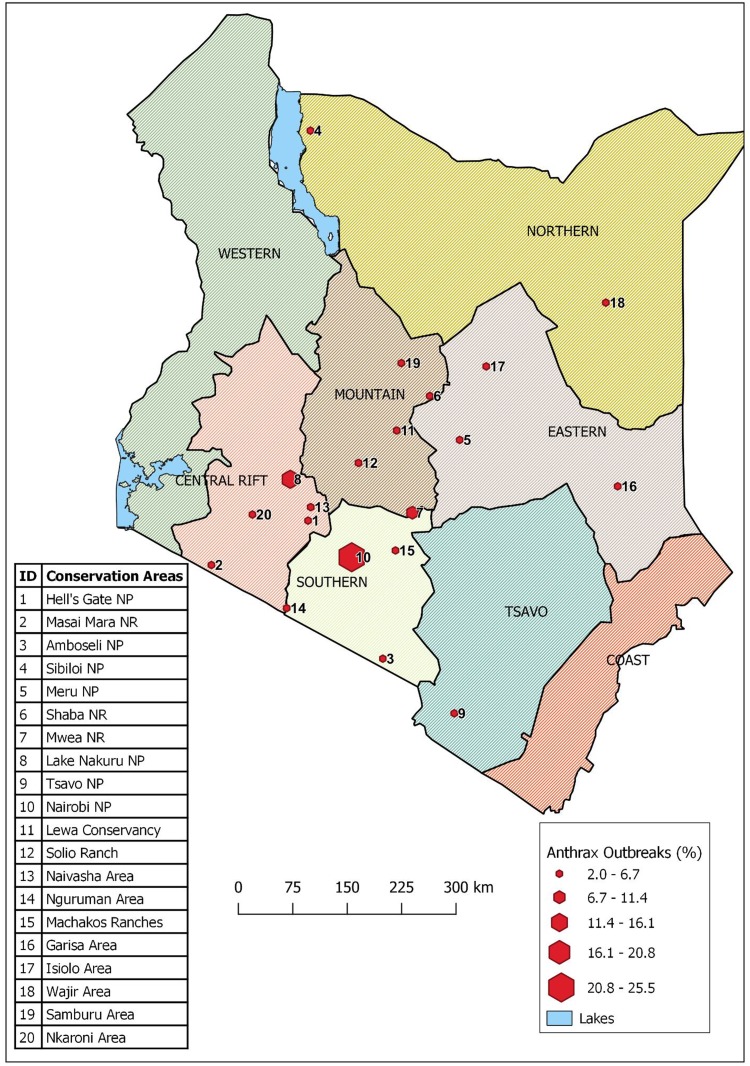
Map of Kenya showing the eight administrative wildlife conservation regions (shaded in different colours) and the conservation areas (red hexagons) where the 51 anthrax outbreaks were reported during 1999–2017. NP refers to national park, NR refers to national reserve.

We developed a database of all the outbreaks in an MS Excel^®^ sheet with the following as variables: conservation region, conservation area, agro-ecological zone (AEZ), month and year of occurrence, wildlife species affected and its food niche. The KWS has administratively divided the country into eight wildlife conservation regions depending on geographical location in Kenya: Coast, Tsavo, Southern, Central Rift, Western, Mountain, Eastern and Northern conservation regions (Fig. 1). Within the eight conservation regions are 60 conservation areas consisting of parks, reserves, sanctuaries and conservancies that contain wildlife that are either in protected areas (secured and monitored national parks, national reserves or national sanctuaries) or unprotected free-ranging areas where they sometimes mingle with domestic animals. AEZs are land units defined based on the patterns of soil, landform and climatic characteristics. Kenya has seven AEZs that include agro-alpine, high potential, medium potential, semi-arid, arid, very-arid and desert. Cross-tabulation of variables was carried out using Pivot tables in Excel and analysis was carried out using the Stata statistical software (Stata12/SE, StataCorp, College Station, TX, USA). We used quantum geographical information system (QGIS) software to create a map showing locations of conservation areas and free-ranging areas for Kenyan wildlife [15].

## Results

### Distribution of anthrax outbreaks and mortalities by conservation areas

Between 1999 and 2017, 51 anthrax outbreaks were recorded in 20 of 60 wildlife conservation areas spread across seven of the eight wildlife conservation regions of the country, with the exception of Western region (Table 1, Fig. 1). Of these, 19 outbreaks (37%) were reported in Nairobi and Lake Nakuru conservation areas, whereas the other 32 outbreaks (63%) occurred across 18 conservation areas. A total of 1014 wild animal deaths associated with the 51 anthrax outbreaks were recorded, with 81% (*N* = 816) of these deaths occurring in the Lake Nakuru conservation area during six separate outbreaks.

**Table 1.** Distribution of anthrax outbreaks and mortalities by conservation area, county and agro-ecological zone

Conservation area	Outbreak numbers (%)	Mortality numbers (%)	County	Dominant agro-ecological zone
Nairobi national park	13 (25.5)	38 (3.7)	Nairobi	Medium potential to semi-arid
Lake Nakuru national park	6 (11.8)	816 (80.5)	Nakuru	Medium potential to semi-arid
Mwea national reserve	5 (9.8)	35 (3.5)	Embu	Semi-arid
Nkaroni area	3 (5.9)	11 (1.1)	Samburu	Arid
Samburu area	3 (5.9)	58 (5.7)	Samburu	Arid
Solio ranch	2 (3.9)	2 (0.2)	Laikipia	Medium potential to semi-arid
Meru national park	2 (3.9)	3 (0.3)	Meru	Medium potential
Hell's Gate national park	2 (3.9)	5 (0.5)	Nakuru	Semi-arid
Maasai Mara national reserve	2 (3.9)	6 (0.6)	Narok	Medium potential
Machakos ranches	2 (3.9)	2 (0.2)	Machakos	Medium potential
Naivasha area	2 (3.9)	12 (1.2)	Nakuru	Semi-arid
Lewa conservancy	1 (2.0)	1 (0.1)	Meru	Medium potential to semi-arid
Isiolo area	1 (2.0)	2 (0.2)	Isiolo	Very arid
Shaba national reserve	1 (2.0)	1 (0.1)	Isiolo	Very arid
Sibiloi national park	1 (2.0)	2 (0.2)	Marsabit	Very arid
Garissa area	1 (2.0)	12 (1.2)	Garissa	Very arid
Nguruman area	1 (2.0)	4 (0.4)	Kajiado	Medium potential to semi-arid
Tsavo	1 (2.0)	2 (0.2)	Taita Taveta/Makueni	Arid
Amboseli national park	1 (2.0)	1 (0.1)	Kajiado	Arid
Wajir area	1 (2.0)	1 (0.1)	Wajir	Very arid
Total	51	1014		

When we defined the dominant AEZ for each conservation area where an anthrax outbreak occurred, we found that the 51 outbreaks were spread across six of the seven AEZs, with the exception of the agro-alpine zone that did not have any reported outbreaks (Table 1).

### Distribution of outbreaks by species

The anthrax outbreaks affected 24 different wildlife species. Of the 51 outbreak records, buffaloes (23.5%), black rhinos (21.6%) and elephants (17.6%) were the most frequently affected species (Table 2). Except for few lions and foxes (two species) that were involved in two outbreaks, the other animal species involved in outbreaks were 22 herbivore species, including 12 (54.5%) with grazers, five (22.7%) with browsers and five (22.7%) with mixed grazers and browsers (Table 2) implying grazer and mixed grazer/browser herbivores were threefold more frequently affected than browser (77% *vs.* 23%).

**Table 2.** Frequency of wildlife species involvement in the anthrax outbreaks and the species food niche

Wildlife species	Number of outbreaks (%)	Number affected
Buffalo (*Syncerus caffer*)	12 (23.5)	760
Black rhino (*Diceros bicornis*)	11 (21.6)	17
Elephant (*Loxodonta africana*)	9 (17.6)	11
Plains zebra *(Equis burchelli*)	4 (7.8)	36
Grevy zebra (*Equus grevyi*)	4 (7.8)	103
Hippopotamus (*Hippopotamus amphibious*)	4 (7.8)	20
Eland (*Taurotragus oryx*)	3 (4.0)	6
White rhino (*Ceratotherium simum*)	3 (5.9)	7
Rothschild giraffe (*Giraffa camelopardalis rothschildi*)	3 (5.9)	13
Warthog (*Phacochoerus aethiopicus*)	3 (5.9)	3
Thompsons gazelle *(Gazella thomsoni)*	2 (3.9)	2
Giraffe (*Giraffa camelopardalis*)	2 (3.9)	11
Bushbuck (*Tragelaphus scriptus*)	2 (3.9)	6
Hartebeest (*Alcelaphus buselaphus)*	1 (2.0)	4
Impala (*Aepyceros melampus*)	1 (2.0)	2
Waterbuck (*Kobus ellipsiprymnus*)	1 (2.0)	1
Gazelle (*Gazella gazelle*)	1 (2.0)	2
Gerenuk (*Litocranius walleri*)	1 (2.0)	1
East African oryx (*Oryx beisa*)	1 (2.0)	1
Topi (*Damaliscus lunatus*)	1 (2.0)	1
Wildebeest (*Connochaetes gnou*)	1 (2.0)	3
Fox (*Vulpes vulpes*)	1 (2.0)	2
Lion (*Panthera leo*)	1 (2.0)	2
Lesser kudu (*Ammelaphus imberbis*)	1 (2.0)	1

#### Temporal and seasonal distribution of outbreaks

**Fig. 2.**
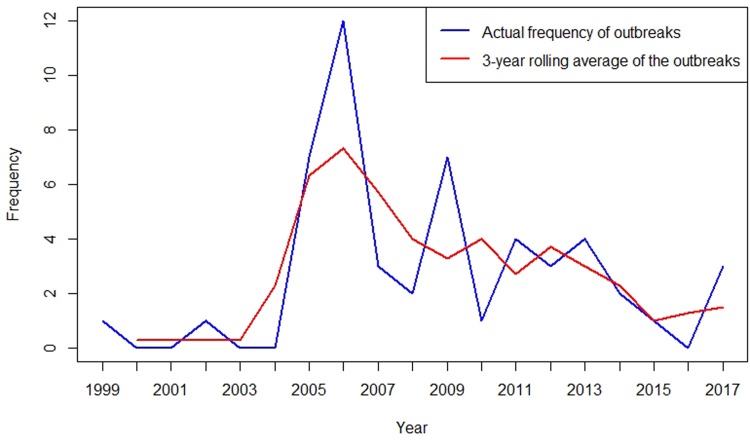
Three-yearly rolling average of anthrax outbreaks in Kenyan wildlife, 1999–2017.

**Fig. 3.**
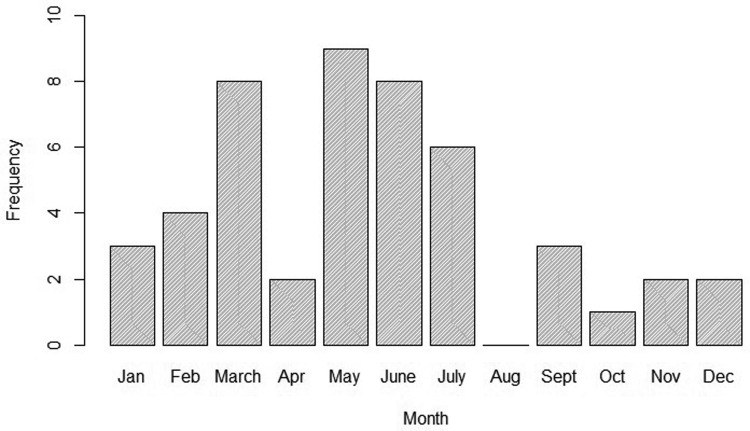
Monthly distribution of anthrax outbreaks in Kenyan wildlife between 1999 and 2017.

To better describe the temporal trend of anthrax outbreaks, a 3-yearly rolling average of the outbreaks from 1999 to 2017 was considered (Fig. 2). The temporal distribution of outbreaks between 1999 and 2017 shows an increase in the frequency of occurrence between 2005 and 2017 (Fig. 2). When classified by month of occurrence, the data show that of 48 outbreaks with information, 32 (67%) occurred during the dry months, including 15 (31%) in the dry and hot season between January to March, and 17 (36%) in the dry and cold months of June to September (Fig. 3). In contrast, 16 (33%) occurred during the rainy seasons including 11 (23%) during the long rain of April and May and five (10%) during the short rains of October to December (Fig. 3).

## Discussion

The findings of this study demonstrated the risk posed by *B. anthracis* to the wildlife population, illustrated by an outbreak at Lake Nakuru national park in 2015 that resulted in the death of 10.5% of all wildlife herbivores in the park, including 17% of the buffalos and 16% endangered black rhinos and white rhinos. Even though outbreaks were reported in <50% of all the wildlife conservation areas in Kenya, we suspected that the outbreaks were more widespread but unreported because of the inherent weaknesses of detecting wildlife deaths in the presence of scavengers.

Our data show that anthrax outbreaks were reported in both dry and wet seasons, however two-thirds of the outbreaks occurred during the 7 months of the dry seasons (January, February, March, June, July, August and September) in Kenya, perhaps suggesting that reduced moisture content is more important than temperature in the occurrence of outbreaks. This finding is consistent with other studies and could be attributed, in a limited way, to increased soil ingestion and spore inhalation during animal feeding when vegetation is depleted [16–21]. It is likely that animals also exhibit increased geophagia (consumption of soil) during the dry seasons, particularly in areas with mineral deficiencies [22]. Additionally, there is a social mixing of animals, both wildlife and domestic animals that invade national conservation areas during the dry seasons in search of pasture and water. The dry season is also linked with stress in animals associated with nutritional deficiencies, heat and parasitism resulting in lowered host immunity [23, 24]. During the dry seasons, vegetation becomes increasingly dry and abrasive and may increase the chances of broken mucosa in the mouth facilitating entry of ingested *B. anthracis* spores into the blood circulation [25]. Receding spore-contaminated water following flooding may also contaminate pastures thus establishing new foci of infection to grazer herbivores. Most wildlife conservation areas in Kenya are located in arid and semi-arid lands, similar to the situation in other countries in sub-Saharan Africa including Namibia, South Africa, Zimbabwe and Tanzania [7, 26–28]. However, it is interesting to note that we did not find records of anthrax outbreaks in wildlife conservancies in agro-alpine regions of Kenya including the Aberdare and Mount Kenya national parks. This observation is consistent with our findings that more outbreaks occur during the dry season, and it may be explained by the similar associated risk factors [12].

The other finding in our study was that >90% of the wildlife species affected by anthrax outbreaks were herbivore, primarily because of their well-documented high susceptibility to *B. anthracis* infection when compared to carnivores and primates [17, 23, 29]. Among herbivores, we found that grazer and mixed grazer/browser herbivores were threefold more frequently affected than browser (77% *vs.* 23%). This finding is in contrast with observations from studies in southern Africa, including in Malilangwe wildlife reserve in Zimbabwe where browsers accounted for >75% of herbivore mortalities, >3-fold higher than that observed in grazers and mixed grazer/browser [7, 30]. The different levels of susceptibility between grazers and browsers are attributed to feeding behaviour; grazers feed closer to the soil where *B. anthracis* spores are maintained thus increasing the risk of ingesting or inhaling the infective spores. However, the higher anthrax occurrence among browsers in southern Africa is attributed to necrophagic flies that transfer the spores from carcasses to high vegetation leaves and twigs that browsers feed on [7, 19, 31, 32].

Apart from acquiring *B. anthracis* from vegetation and soil during feeding, it is suspected that the behaviour of wildlife associated with caring for the sick and dying may lead to exposure [33, 34]. Certain herbivore species, including buffaloes, tend to sniff and lick carcasses of their own species, increasing the chance of ingesting or inhaling anthrax spores. This mechanism may have played a major role during an anthrax outbreak in Lake Nakuru National Park in 2015 where >750 buffalos, constituting 17% of the species population at that national park died of anthrax in one outbreak [12]. Some studies have also suggested that certain *B. anthracis* strains may have a higher predilection and/or virulence towards certain host species [23]. This is noteworthy considering that few genotypic studies of *B. anthracis* have been carried out in sub-Saharan Africa [35].

This study had some limitations. Isolated anthrax deaths in wildlife may go undetected because of quick action of scavengers to clear the carcass, and because the outbreak occurred in remote inaccessible locations. Molecular methods were not used to confirm the presence of *B. anthracis*; therefore, it is possible that microscopy may have misidentified other *Bacillus* spp (e.g. *B. cereus* biovar *anthracis*) as *B. anthracis.* However, no other *Bacillus* spp has been associated with widespread die-offs in mammals nor reported as a contaminant in blood in characteristically large numbers in anthrax deaths in Kenya to invalidate the conclusions.

Buffaloes (23.5%), black rhinos (21.6%) and elephants (17.6%) were the most frequently affected species. Our findings demonstrate the extensive geographic distribution of wildlife anthrax in the country, making it one of the important infectious diseases that threaten wildlife conservation.

In conclusion, our findings reveal the widespread geographic distribution of wildlife anthrax in Kenya, mostly affecting the large mammals important in conservation efforts. These findings underscore the need for greater empirical attention consisting of better wildlife disease surveillance and research, perhaps seasonally focused, for early detection of anthrax and control programmes.
